# Association of Ultrasonography With Final Histopathology in Diagnosing Thyroid Malignancy: A Single-Institute Retrospective Study

**DOI:** 10.7759/cureus.31677

**Published:** 2022-11-19

**Authors:** Ali S Alshahrani, Montasir Junaid, Abdulrahman A Aldosari, Khaled A Amer, Ali M Al Qannass

**Affiliations:** 1 Otolaryngology - Head and Neck Surgery, Armed Forces Hospital Southern Region, Khamis Mushait, SAU; 2 College of Medicine, King Khalid University, Abha, SAU

**Keywords:** ti-rads, thyroid, correlation, concordance, histopathology, ultrasound, lesion, thyroid nodule

## Abstract

Background

Thyroid nodules are well-defined regions of aberrant echogenicity within the thyroid parenchyma that are radiologically distinct from the normal thyroid gland. The most common incidental finding in imaging scans that include the neck is a thyroid nodule. Rarely are thyroid nodules cancerous, as the majority are benign.

Aim

The current study aims to assess the concordance between ultrasound (US) of thyroid nodules and final histopathology results to identify the different types of detected thyroid lesions.

Methodology

A retrospective study reviewed the medical files of all patients presenting to the Armed Forces Hospital, Southern Region, with suspected thyroid nodules from April 2018 to January 2020. Data were extracted using pre-structured proforma to avoid inconsistency. Data extracted included patient demographic, swelling laterality, size, and US and histopathological findings.

Results

In the present study, 47 samples had a mean age of 44.27 (SD = ±13.5) years, 85.1% were of the female gender, the majority (85.1%) had multiple nodules, 38.3% were with Thyroid Imaging Reporting and Data System (TI-RADS) TR4 US score, and the median size of the nodule on US was 3 cm with a range of 0.6 to 14 cm. The study showed that 10% of TR1 samples were lymphocytic in histopathology, 66.7% of TR3 samples were benign multinodular goiter in histopathology, and 55.6% of samples of TR4 were malignant in histopathology.

Conclusions

The current study showed that the malignancy rate of the examined nodules was not uncommon both by US and histopathology, where papillary carcinoma was the most detected malignancy. The study showed a satisfactory agreement rate between TI-RADS classification by US sonography and histopathological reporting, where TR4 and TR5 by the US were mainly categorized as pre-malignant/malignant lesions by histopathology.

## Introduction

A thyroid nodule is a characteristic lesion found incidentally on computed tomography (CT) scans and magnetic resonance imaging (MRI). It is considered a common finding in the general population [1]. The rate of accidental thyroid nodules is believed to be up to 50% [2]. Most thyroid nodules are benign, while malignant nodules are detected in 3-7% of patients [3]. The diagnosis of thyroid nodules continues to present significant challenges for treating physicians. The frequent use of high-resolution ultrasound (US) in conjunction with carotid Doppler techniques revealed the existence of an increasing number of incidentalomas or asymptomatic thyroid nodules [4]. The sensitivity of the US is high for identifying the nodules, and the US characteristics of the nodules can help determine whether further investigation is required [5]. Moreover, ultrasound-guided fine-needle aspiration biopsy (US-FNAB) is currently highly recommended [6]. Real-time ultrasonography provides continuous imaging of needle insertion and sample collection, making it possible to verify that the needle is within the lesion with ease and assurance. Small suspicious nodules that are solid and cystic can be identified and carefully biopsied using a needle that can be guided to the solid areas [7,8].

Solitary nodule patients had a higher risk of thyroid cancer than those with numerous nodules, according to Li et al. [9]. Before, multinodular goiters were thought to be benign and had a low chance of being malignant [10]. Multinodular goiters should be treated as a single nodule, according to Gandolfi et al. [11]. In addition, El-Gammal et al. discovered that multinodular goiters were statistically more likely to be malignant than single thyroid nodules [12]. Thyroid nodules have been studied for benign and malignant sonographic characteristics [13,14]. The Thyroid Imaging Reporting and Data System (TI-RADS) have a standardized scoring system that looks at the composition, margin, echogenicity, shape, margin, and echogenic foci of the thyroid ultrasonogram. TI-RADS is a scoring system of five different categories (TR1-TR5), with higher levels indicating an increased probability of malignancy and hence more focused clinical management [15].

US-FNAB has become the preferred approach for assessing thyroid nodules due to its extensive convenience. Nondiagnostic and false-negative results can be reduced by using US-FNAB on a routine basis [16,17]. The current study aimed to assess the concordance between US and final histopathology for a thyroid nodule to identify the different types of detected thyroid lesions.

## Materials and methods

A retrospective study was conducted by reviewing electronic medical files (e-files) of all patients presenting to the Armed Forces Hospital, Southern Region, with thyroid nodules from April 2018 to January 2020. Any cases with neck swelling other than thyroid swellings were excluded. Also, medical files with incomplete/missing relevant data were excluded. There was no contact or risk to patients since the data were extracted from electronic patient files from the last three years, and all patients have already been operated/treated upon their condition. Data were extracted using pre-structured proforma to avoid inconsistency. Data gathered included patient demographics, swelling laterality, size, and US and histopathological findings.

Statistical analysis

IBM SPSS version 23.0 (IBM Corp., Armonk, NY) was used to store and analyze data. For baseline patient characteristics, US results, fine-needle aspiration cytology (FNAC), histopathology findings, and other examined parameters of the cohort, and counts with percentages were provided. Mean with standard deviation or median with range were given for quantitative measures. The relationship between US findings, FNAC, and histology was examined using Pearson's chi-square at a 5% significance level. Figures were also utilized to show data graphically.

## Results

Table 1 summarizes the baseline characteristics of the studied samples. In the present study, 47 samples had a mean age of 44.27 (SD = ±13.5) years, 85.1% were of the female gender, the majority (85.1%) had multiple nodules, 38.3% were with TR4 US finding score, and the median size of the nodule on US was 3 cm with a range of 0.6 to 14 cm on average.

**Table 1:** Descriptive statistics on gender, neck swelling, and ultrasound findings TI-RADS: Thyroid Imaging Reporting and Data System.

Variables		n	%
Gender	Female	40	85.1
	Male	7	14.9
Age (years)	Mean ± SD	44.27	±13.5
Neck swelling	Single nodule	7	14.9
	Multiple nodules	40	85.1
Ultrasound findings: TI-RADS score	TR1	10	21.3
	TR2	12	25.5
	TR3	6	12.8
	TR4	18	38.3
	TR5	1	2.1
Size of the nodule in cm on ultrasound or the largest nodule in multinodular goiter	Median (range)	3.0 cm	0.6-14 cm

Table 2 reports that 80.9% of samples had a total thyroidectomy procedure, 40.4% had a papillary type of malignancy, and 57.4% had the largest nodule on the right side.

**Table 2:** Outcomes on the procedure, type of malignancy, and side of the largest nodule Ca: carcinoma.

Parameters		n	%
Procedure done	Total thyroidectomy	38	80.9
	Thyroidectomy with neck dissection	7	14.9
	Partial thyroidectomy	2	4.3
Type of malignancy	N/A	25	53.2
	Papillary Ca	19	40.4
	Follicular Ca	1	2.1
	Medullary Ca	1	2.1
	Lymphoma	1	2.1
Side of the largest nodule	Right	27	57.4
	Left	20	42.6

**Figure 1:**
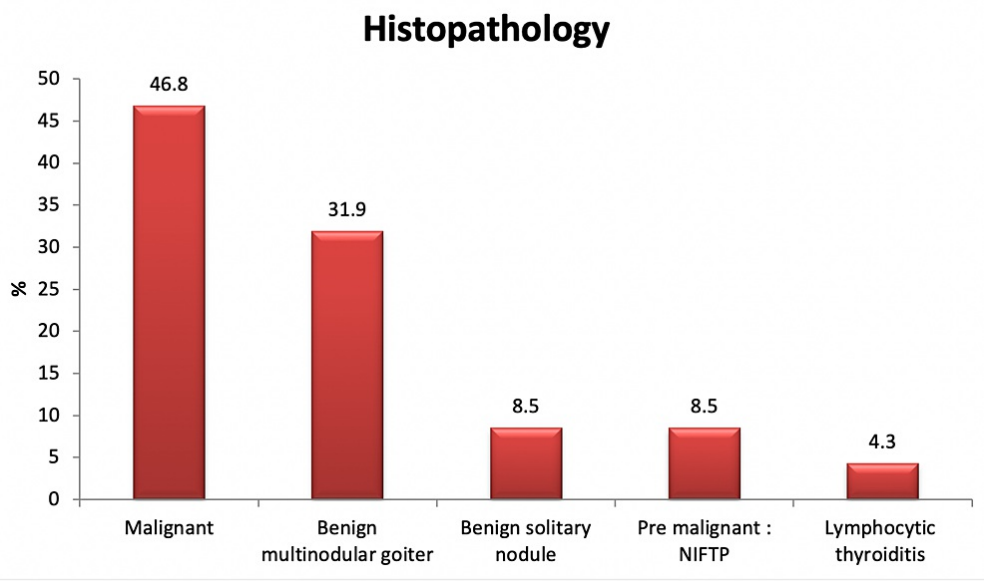
Final histopathology result NIFTP: non-invasive follicular thyroid neoplasm with papillary-like nuclear features.

Table 3 shows the outcomes of histopathology. Of the samples, 31.9% were found with benign multinodular goiter and 46.8% were malignant. Figure 1 provides a detailed presentation of histopathology outcomes.

**Table 3:** Histopathology final result NIFTP: non-invasive follicular thyroid neoplasm with papillary-like nuclear features.

Histopathology (final result)	n	%
Lymphocytic thyroiditis	2	4.3
Benign multinodular goiter	15	31.9
Benign solitary nodule	4	8.5
Premalignant: NIFTP	4	8.5
Malignant	22	46.8

Table 4 reports the association between US results and histopathology outcomes. Results showed that 10% of TR1 samples were lymphocytic in histopathology, 66.7% of TR3 samples were benign multinodular goiter in histopathology, and 55.6% of TR4 samples were malignant in histopathology. Pearson's chi-square test did not indicate a significant association between US findings and the final results of histopathology (p = 0.269).

**Table 4:** Association between ultrasound and histopathology for thyroid nodules P = 0.269 using Pearson's chi-square test. TI-RADS: Thyroid Imaging Reporting and Data System; NIFTP: non-invasive follicular thyroid neoplasm with papillary-like nuclear features.

Histopathology (final result)	Ultrasound findings: TI-RADS score									
	TR1		TR2		TR3		TR4		TR5	
	n	%	n	%	n	%	n	%	n	%
Lymphocytic thyroiditis	1	10.0	0	0.0	0	0.0	1	5.6	0	0.0
Benign multinodular goiter	3	30.0	3	25.0	4	66.7	5	27.8	0	0.0
Benign solitary nodule	0	0.0	3	25.0	0	0.0	1	5.6	0	0.0
Premalignant: NIFTP	3	30.0	0	0.0	0	0.0	1	5.6	0	0.0
Malignant	3	30.0	6	50.0	2	33.3	10	55.6	1	100.0

## Discussion

The thyroid gland may be seen with US, a diagnostic imaging modality that applies high-frequency sound waves [18]. Ultrasonography of the thyroid gives the most accurate information about the shape and structure of nodules [19]. They are commonly used by doctors to distinguish between cysts and solid nodules or to determine how many nodules are present. It is also possible that they will use it as a guide for a fine-needle aspiration biopsy [20]. It is crucial to note that no single US characteristic can be used to distinguish benign from malignant nodules [21].

The current study aimed to assess the US of thyroid nodule correlation with final histopathology. The study results showed that more than one-third of the thyroid nodules by US were TR4, while less than half of the nodules were TR1 and TR2. The average size detected by the US was 3 cm, with a range of 0.6-14 cm. Histopathology showed that malignant nodules were dominant (46.8%), followed by benign multinodular goiter (about one-third of the nodules). Papillary carcinoma was the most diagnosed malignancy among examined nodules. Gul et al. found that margin irregularity, hypoechoic pattern, and microcalcification indicate malignancy at the thyroid nodule based on ultrasonography [22]. The rate of cancer found by the histopathologic exam was 7.6%, which is much lower than the rate estimated by this study. Many studies show that microcalcifications are more common in malignant nodules than benign ones [8,23]. Papini et al. and Koike et al. found that malignant thyroid nodules usually have irregular margins [24,25]. Furthermore, some investigations have found that hypoechoic texture is associated with a greater probability of malignancy [13,26].

This study involves patients who already had a total or partial thyroidectomy, and the surgeon may have already decided that these thyroid nodules were more likely to be cancerous. The strength of the current study is that it only looked at thyroid nodules that had already been removed. In addition, the final histopathology was utilized as the reference point since it provides the highest level of diagnostic certainty. Previous studies that compared how well different US diagnostic guidelines worked only looked at nodules diagnosed based on cytologic features [27,28]. Because of this, some cancerous nodules may be missed, leading to a bias in the results. This study is the only one in the area that uses established US risk stratification systems to look at the US features of thyroid nodules in a systematic way.

Regarding the correlation between US findings and histopathology of the thyroid nodules, the study results showed that 10% of TR1 samples were lymphocytic in histopathology, two-thirds (66.7%) of TR3 samples were benign multinodular goiter in histopathology, and more than half (55.6%) of TR4 samples were malignant in histopathology with no significant association. Cavallo et al. [29] established that 26% of thyroid nodules were cancerous. The highest malignancy rate was found in nodules less than 2 cm in size (30%). With increasing size, 57% of 1 cm nodules were suspicious for malignancy compared to 20% of nodules greater than 6 cm. Smaller nodules exhibited higher malignancy rates than larger nodules at 2, 3, 4, and 5 cm. According to Deveci et al., there is a 50% agreement between an ultrasound and a surgical pathology exam on whether a nodule is benign or malignant, except for small nodules (1.0 cm, 78.5%) [30]. The researchers investigated 133 nodules from 131 individuals who had thyroid surgery for cytologically suspicious, Afirma-suspicious lesions. Thyroid imaging sensitivity was 71.4%, specificity was 38.1%, positive predictive value was 40.2%, negative predictive value was 69.6%, and accuracy was 50.4%.

Our study has a few limitations. First, the study was a single institute-based retrospective analysis of thyroid swellings with a relatively small sample size; however, it might portray the incidence of the disease in our area. We were forced to exclude quite a few patients due to incomplete data (exclusion criteria). Another limitation was the high number of multinodular goiters, which might affect the subjective analysis via ultrasonography of these neck swellings using the TI-RADS scoring system, and a similar effect could be anticipated in performing FNAC in these cases where there is a possibility that the aspiration was not done on the representative lesion.

## Conclusions

The current study showed that the malignancy rate of the examined nodules was not uncommon both by US and histopathology, where papillary carcinoma was the most detected malignancy. Regarding concordance between radiological and histopathological assessment of thyroid nodules, the study found a satisfactory agreement rate between TI-RADS classification by the US and histopathological reporting, with TR4 and TR5 classified as pre-malignant/malignant lesions by histopathology. This study supports the evidence for the crucial significance of US sonography in diagnosing thyroid nodule type and mapping the action necessary in the future. Also, US characteristics and nodule size may achieve satisfactory diagnostic precision.
